# Systematic review of the relationships between combinations of movement behaviours and health indicators in the early years (0-4 years)

**DOI:** 10.1186/s12889-017-4851-1

**Published:** 2017-11-20

**Authors:** Nicholas Kuzik, Veronica J. Poitras, Mark S. Tremblay, Eun-Young Lee, Stephen Hunter, Valerie Carson

**Affiliations:** 1 Faculty of Physical Education and Recreation, University of Alberta, Van Vliet Complex, 8840-114 Street, Edmonton, AB T6G 2H9 Canada; 2 Healthy Active Living and Obesity Research Group, Children’s Hospital of Eastern Ontario Research Institute, Ottawa, ON K1H 8L1 Canada

**Keywords:** Physical activity, Sedentary behaviour, Sleep, Movement behaviours, Adiposity, Motor development, Growth, Fitness, Early years

## Abstract

**Background:**

A recent review highlighted important relationships between combinations of movement behaviours (i.e., sleep, sedentary behaviour, and physical activity) and health indicators among school-aged children and youth (aged 5-17 years). It is unclear whether similar relationships exist in younger children. Therefore, this review sought to examine the relationships between combinations of movement behaviours and health indicators in the early years (1.00 month to 4.99 years).

**Methods:**

Medline, EMBASE, PsycINFO, and SportDiscus were searched for relevant studies up to November 2016, with no date or study design limits. Included studies met the a priori-determined population (apparently healthy children aged 1.00 month to 4.99 years), intervention (combination of ≥2 movement behaviours [i.e., sleep and sedentary behaviour; sleep and physical activity; sedentary behaviour and physical activity; and sleep, sedentary behaviour, and physical activity]), comparator (various levels and combinations of movement behaviours), and health outcome/indicator (Critical: adiposity, motor development, psychosocial health/emotional regulation, cognitive development, fitness, and growth; Important: bone and skeletal health, cardiometabolic health, and risks). For each health indicator, quality of evidence was assessed by study design using the Grading of Recommendations Assessment, Development, and Evaluation (GRADE) framework.

**Results:**

Ten articles (*n* = 7436 participants; *n* = 5 countries) were included. Across observational and experimental study designs, the most ideal combinations of sedentary behaviour and physical activity were: favourably associated with motor development and fitness among preschool-aged children (3.00 to 4.99 years); both favourably and not associated with adiposity among toddlers (1.10 to 2.99 years) and preschool-aged children; and not associated with growth among toddlers and preschool-aged children. The most ideal combinations of sleep and sedentary behaviour were favourably associated with adiposity among infants (1.00 month to 1.00 years) and toddlers. Quality of evidence ranged from “very low” to “moderate”.

**Conclusions:**

The most ideal combinations of movement behaviours (e.g., high sleep, low sedentary behaviour, high physical activity) may be important for optimal health in the early years. Findings can help inform movement behaviour guidelines for the early years. Given the limited evidence, future research is needed to determine the ideal distribution of daily movement behaviours for optimal health throughout the early years.

**Electronic supplementary material:**

The online version of this article (10.1186/s12889-017-4851-1) contains supplementary material, which is available to authorized users.

## Background

The movement behaviour continuum progresses from no- or low-intensity movement to high-intensity movement and encompasses sleep, sedentary behaviour, and physical activity (PA). Novel insights suggest the entire movement behaviour continuum in a 24-h period should be targeted for the optimal health of children [1]. The consideration of all movement behaviours builds upon the traditionally strong emphasis on the health benefits of moderate- to vigorous-intensity physical activity (MVPA) in children. While MVPA is undoubtedly an important health behaviour, it only accounts for a small portion of the day (i.e., 24-h period), whereas light-intensity physical activity (LPA), sedentary behaviour (SB), and sleep make up the majority of the 24-h period [2]. Therefore, targeting all movement behaviours over the 24-h period allows for more opportunities to improve the health of children.

The recent paradigm shift toward a broadened focus on all movement behaviours for improved health has been followed by an accumulating, yet still limited, body of research in the pediatric population (0-18 years) [3–6]. Additionally, advances in the analysis of movement behaviours and health across age groups is growing [7, 8]. As a reflection of these trends, in 2016, Canada released the world’s first *24-Hour Movement Guidelines for Children and Youth* (5-17 years), which provide recommendations for movement behaviours over the course of a 24-h period [9]. One piece of evidence that helped to inform these guidelines was a systematic review of the combinations of physical activity, sedentary behaviour, and sleep, and their relationships with health indicators in school-aged children and youth (5-17 years) [10]. The authors found that school-aged children and youth who had combinations of high physical activity, low sedentary behaviour, and high sleep had more desirable measures of adiposity and cardiometabolic health [10].

The conclusions from the school-aged children and youth review may not apply to children in the earlier years of life because dramatic differences exist between the two age groups in growth and development, which impact movement behaviour patterns [11]. For instance, children in the early years (1.00 month to 4.99 years) need considerably more sleep than school-aged children and youth [12]. Additionally, typically developing early years children will have different movement behaviour patterns as they progress through key movement behaviour milestones such as sleep consolidation [12], initial interests in screen time [13, 14], and initiation of ambulation [15]. Given the changes to movement behaviour patterns, the relationships between movement behaviour compositions and health indicators may also change as a function of a child’s growth and development. Therefore, in order to inform future guidelines and research, it is important to systematically review the evidence for the relationships between combinations of movement behaviours and health indicators in the early years. Specifically, the purpose of this systematic review was to examine the relationships between the combinations of: sleep and sedentary behaviour; sleep and physical activity; sedentary behaviour and physical activity; and sleep, sedentary behaviour, and physical activity; with health indicators in the early years.

## Methods

### Protocol and registration

This systematic review was registered with the International Prospective Register of Systematic Reviews (PROSPERO; Registration no. CRD42016045374; available from http://www.crd.york.ac.uk/PROSPERO/display_record.asp?ID=CRD42016045374), and was conducted and reported following the Preferred Reporting Items for Systematic Reviews and Meta-Analyses (PRISMA) statement [16].

### Eligibility criteria

Articles were included in this review if they were peer-reviewed, written in English or French, published or in press, and met the a priori determined Population, Intervention, Comparator, and Outcome (PICO) study criteria [17]. Case-studies and case series were not included. Grey literature was not included, except for unpublished evidence from clinical trials registries. Studies using predictive modelling (e.g., isotemporal substitution, compositional analysis substitution) were included only if no other data were available for the health indicator. If several predictive models were found for a health indicator, one model was chosen based on expert consensus. The rationale for this decision was that inferences of a predictive model are based on a set of assumptions. Therefore, the certainty of the conclusions from predictive models is presumed to be lower than a model using directly measured evidence [18].

#### Population

The population was apparently healthy children aged 1.00 month to 4.99 years, including those with overweight/obese weight statuses; samples with a diagnosed medical condition were ineligible. If an age range was reported in lieu of the mean, studies with ranges at an upper limit of 5.00 years were included (e.g., 2.00-5.00 years), but studies with ranges at an upper limit of >5.00 were not (e.g., 2.00-6.00 years). If mean age or age range was not reported, samples described as infants, toddlers, preschool-aged children, or pre-elementary were included, but not kindergarteners or older. When possible, results were intended to be examined separately for infants (1.00 month to 1.00 year), toddlers (1.10 to 2.99 years), and preschool-aged children (3.00 to 4.99 years).

#### Intervention (exposure)

For experimental studies, interventions had to target two or three movement behaviours exclusively (e.g., both physical activity and sedentary behaviour, but no diet component targeted). However, an observed change in the targeted behaviours was not necessary. For observational studies, the exposure was any combination of two or three movement behaviours (i.e., sleep, sedentary behaviour, and physical activity). Briefly, relevant interventions/exposures for each individual movement behaviour were operationalized as the durations (sleep, sedentary behaviour, and physical activity), patterns and types (sedentary behaviour and physical activity), and intensities (physical activity) of behaviours. Full definitions for each movement behaviour are described elsewhere [19–21].

#### Comparator

The comparator was various durations and combinations of movement behaviours. However, a comparator group or control group was not required for inclusion.

#### Outcomes (health indicators)

The outcomes were nine health indicators from the PROSPERO registrations of three individual movement behaviour reviews in the early years (i.e., sleep [PROSPERO 2016: CRD42016040096], sedentary behaviour [PROSPERO 2016: CRD42016035270], and physical activity [PROSPERO 2016: CRD42016035937]), which were agreed upon by the review team and collaborators. Health indicators for each individual review were selected and ranked as either “critical” or “important” by expert consensus following the Grading of Recommendations Assessment, Development, and Evaluation (GRADE) framework [18]. To be ranked as “critical” in this review, the health indicator had to be ranked critical in at least one of the other reviews, while all other indicators were ranked as “important”. Critical health indicators were: adiposity (e.g., overweight, obesity, body mass index [BMI], skinfold thickness, body fat, waist circumference), motor development (e.g., gross motor skills, fine motor skills, locomotor and object control), psychosocial health/emotional regulation (e.g., self-efficacy, self-esteem, prosocial behaviour, aggression, social functioning, depressive symptoms, anxiety symptoms, quality of life, stress, mood, hyperactivity/impulsivity), cognitive development (e.g., language development, attention, executive functioning), fitness (e.g., cardiovascular fitness, musculoskeletal fitness), and growth (e.g., height/length, weight, head circumference). Important health indicators were: bone and skeletal health (e.g., bone mineral content, bone density), cardiometabolic health (e.g., blood pressure, glucose, insulin resistance, blood lipids), and risks (e.g., injury/harm).

### Information sources and search strategy

The search strategy for the current review was developed by combining the search strategies from the three individual movement behaviour reviews [19–21]. The search strategies for the individual reviews were created and peer-reviewed by two librarians with expertise in systematic reviews. The following databases were searched between July 29 and August 9, 2016: SPORTDiscus (July 29, 2016), MEDLINE (August 9, 2016), Embase (August 9, 2016), and PsycINFO (August 9, 2016). No date or study design limits were included. Additionally, a search update was conducted in all databases on November 1, 2016 (see Additional File 1 for the complete search strategies).

All records retrieved from the database searches were imported into Reference Manager Software (Version 11, Thompson Reuters, San Francisco, CA, USA), in the following order: MEDLINE, Embase, PsycINFO, and SportDiscus. After each database was added, any duplicate records were removed. Records were then imported into DistillerSR Software (Evidence Partners, Ottawa, ON, Canada) to begin the screening process. All aspects of the screening process were led by one reviewer and, where applicable, a second reviewer repeated the screening process. More specifically, titles and abstracts were first screened by one reviewer and, if excluded, were then screened by another reviewer. Records included by at least one reviewer were obtained for further screening. Next, full-text articles were obtained and first screened by one reviewer, and then screened by another reviewer. Inclusion or exclusion of an article required agreement by both reviewers. Any disagreements between the two reviewers were resolved by a third reviewer or the review team.

Review articles were also flagged during the screening process, and reference lists were manually searched. Finally, two trial registries (https://clinicaltrials.gov and http://www.who.int/ictrp/en/) were searched using the following terms: “physical activity” “sedentary behaviour/behavior”, “sleep”, and “child/infant/toddler/early childhood” on December 22, 2016.

### Data extraction

Data were extracted from each included article into Microsoft Excel. These data included descriptive information, relevant exposure and health indicator details, as well as study results. When unadjusted and adjusted results were presented, extraction was completed for the unadjusted model and the most fully adjusted model. Furthermore, for this review, statistical significance was defined as a *p*-value of less than 0.05 regardless of how significance was defined in individual articles. One reviewer completed data extraction for each included article; extracted data was then verified by an additional reviewer.

### Quality assessment

The GRADE framework was used to assess the collective quality of evidence across studies by health indicator and study design [22]. Quality of evidence was rated as “very low”, “low”, “moderate”, or “high” based on five criteria: risk of bias, inconsistency, indirectness, imprecision, and other (e.g., dose-response evidence). All studies started at a “low” quality of evidence rating, except randomized controlled trials, which started at a “high” rating. Quality of evidence was downgraded for any study design if there were limitations in any of the five criteria. If no downgrading occurred, non-randomized and observational study designs could be upgraded to “moderate” or “high” quality of evidence if large or very large effect sizes and/or dose-response evidence were present. However, dose-response evidence cannot be determined for cross-sectional studies, so the quality of evidence in these studies was upgraded only if there was a gradient of higher exposure with higher/lower health indicator.

At the individual study level, only risk of bias was assessed. For experimental studies, the risk of bias was assessed using methods described in the Cochrane Handbook [23]. For observational studies, the risk of selection bias, performance bias, selective reporting bias, detection bias, attrition bias, and other biases (e.g., inadequate control for key confounders) were assessed [24]. When the only sources of bias were performance bias due to lack of intervention/control group blinding or selection bias due to convenience sampling, this was not considered “serious risk of bias”. Risk of bias was assessed by one reviewer for all included articles, and then verified by another reviewer. One reviewer then evaluated overall quality of evidence, which was verified by the review team.

### Data analysis

Meta-analyses were planned for sufficiently homogeneous data in terms of statistical, clinical, and methodological characteristics using Review Manager Software 5.3 (The Cochrane Collaboration, Copenhagen, Denmark). However, based on high levels of heterogeneity in study design as well as exposure and outcome variables, meta-analyses were not appropriate. Consequently, a narrative synthesis was conducted, with all studies weighted equally and structured by health indicator, study design, and combination of movement behaviours (e.g., sleep and sedentary behaviour). Due to the heterogeneity in exposure variables across studies, “most ideal combinations of movement behaviours” was used as an overarching summary term to reflect any combination of movement behaviour hypothesized to be beneficial for health based on older populations (e.g., high sleep, low sedentary behaviour, and high physical activity; increased sleep, decreased sedentary behaviour, increased physical activity; sedentary behaviour replaced with physical activity) [10, 19, 25, 26].

## Results

### Description of studies

**Fig. 1:**
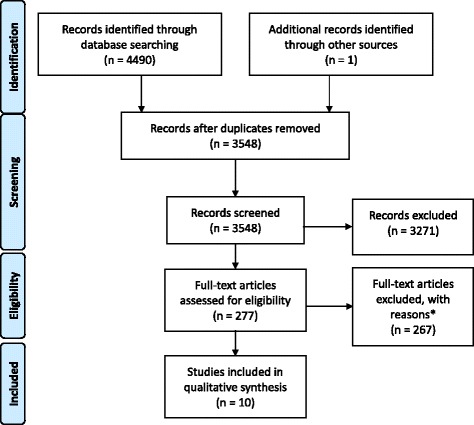
PRISMA flow-chart of included studies. *Articles were excluded for: not being original research (n=8), participants not being of the early years (n=102), not being apparently healthy (n=7), not reporting a combination of movement behaviours (n=28), not reporting the relationship between movement behaviours and a health indicator (n=119), observational studies with exposures combining movement behaviours with other behaviours (e.g., diet) (n=4), intervention studies not exclusively targeting movement behaviours (e.g., targeting sleep, physical activity, and diet) (n=4). Some articles were excluded for more than one of the above reasons

A total of 4490 records were found in the database search and one record was found by manually searching reference lists. After de-duplication, 3548 records remained. After title and abstract screening, 277 full-text articles remained, of which 10 articles met inclusion criteria. A summary of reasons for excluding articles during full-text screening is presented in Fig. 1. No additional articles were identified through trial registry searches.

The 10 included studies (nine unique samples) had a total of 7436 participants (7353 unique participants). Mean ages ranged from 3.29-4.97 years, including infants and toddlers in 1/10 studies, toddlers and preschool-aged children in 3/10 studies, and preschool-aged children alone in 6/10 studies. Studies represented data from five countries (Canada *n* = 3, Greece *n* = 1, the United States *n* = 4, Scotland n = 1, and Sweden n = 1), and were published between 2006 and 2016. Four studies used an experimental design (cluster randomized controlled trial [RCT] n = 3, non-randomized intervention n = 1), and six studies used an observational design (cross-sectional n = 4, longitudinal *n* = 2). Of the combinations of movement behaviours in the literature, 9/10 studies involved sedentary behaviour and physical activity, and 1/10 studies involved sleep and sedentary behaviour. No other combinations were found. Movement behaviours were assessed using accelerometers in six studies [27–32] and parental-report questionnaires in four studies [33–36]. Based on the heterogeneous statistical, clinical, and methodological characteristics within the included studies, a meta-analysis was not performed, but a narrative synthesis was conducted. For a full list of study characteristics and results, see Additional File 2: Tables S1-S4.

### Data synthesis

#### Adiposity

The associations between combinations of movement behaviours and adiposity were examined in eight studies (see Table 1 and Additional File 2: Table S1). All studies included a height-for-weight indicator of adiposity (e.g., BMI percentile); 4/8 studies classified children according to weight status (e.g., normal weight, overweight, obese); 1/8 studies measured skinfolds; and 1/8 studies measured body fat via bio-electrical impedance. Combinations of movement behaviours included sedentary behaviour and physical activity in 7/8 studies, and sleep and sedentary behaviour in 1/8 studies. Studies included experimental designs (3 studies) and observational designs (5 studies).

**Table 1:** The relationship between movement behaviours and adiposity

No. of studies	Design	Quality assessment					No. of participants	Absolute effect	Quality
		Risk of bias	Inconsistency	Indirectness	Imprecision	Other			
Mean baseline ages ranged from 3.29-4.97 years. One study had exposure measurements as early as 6 months (no average provided) but averaged several exposure measurements over 2 years. Data were collected by clustered RCT (*n* = 2), non-randomized intervention (*n* = 1), longitudinal (*n* = 2), and cross-sectional (*n* = 3) study designs. All height-for-weight indices of adiposity were objectively measured except in one study, which did not clearly indicate how measurement occurred. Other indicators of adiposity were assessed via bioelectrical impedance, and skinfold thickness (subscapular and tricep surae).									
2	Cluster RCT^a^	Serious risk of bias^b^	No serious inconsistency	Serious indirectness^c^	No serious imprecision	None	1245	SB + PA: The movement behaviour interventions were *not* associated with changes in weight-for-height indices in 2 studies [27, 28]. The movement behaviour intervention was *favourably* associated with body fat in 1 study [28].	LOW^d^
1	Non-randomized intervention^e^	Serious risk of bias^f^	No serious inconsistency	No serious indirectness	No serious imprecision	None	86	SB + PA: The movement behaviour intervention was *favourably* associated with BMI reduction in toddlers, but *not* in the preschool-aged sample [29].	VERY LOW^g^
2	Longitudinal^h^	Serious risk of bias^i^	No serious inconsistency	Serious indirectness^j^	No serious imprecision	Dose-response & large magnitude of an effect ^k^	1827	SB + PA: Classification based on SB + PA variables was *not* associated with BMI percentile over 2 years in 1 study [30]. SLEEP + SB: High levels of sleep and low levels of TV time were *favourably* associated with BMI-z scores, sum of skinfold thickness, and overweight status, and *not* associated with skinfold thickness ratio when compared to low levels of sleep and high levels of TV time in 1 study [36]. High levels of sleep and low levels of TV time were *favourably* associated with BMI-z score, and *not* associated with sum of skinfold thickness, overweight status, and skinfold thickness ratio when compared to low levels of sleep, and low levels of TV time in 1 study [36]. High levels of sleep and low levels of TV time were *not* associated with BMI-z score, sum of skinfold thickness, overweight status, and skinfold thickness ratio when compared to high levels of sleep and high levels of TV time in 1 study [36].	VERY LOW^l^
3	Cross-sectional^m^	Serious risk of bias^n^	No serious inconsistency	No serious indirectness	No serious imprecision	None	3888	SB + PA: Children with high amounts of SB and low amounts of PA were *favourably* associated with obesity classification in 1 study [33] and *not* associated with obesity classification in 2 studies [34, 35].	VERY LOW^o^

*BMI* body mass index, *CI* confidence interval, *LPA* light-intensity physical activity, *MET* metabolic equivalent, *MVPA* moderate- to vigorous-intensity activity, *OR* odds ratio, *PA* physical activity, *RCT* randomized controlled trial, *SB* sedentary behaviour, *TPA* total physical activity, *TV* television

^a^ Includes 2 cluster RCTs [27, 28]

^b^ Serious risk of bias. In 1 study, age was not adjusted for in the analysis [28]

^c^ Serious indirectness. The sedentary behaviour component of the intervention was minimal in both studies, which could have caused a risk for indirectness. However, in 1 study the intervention significantly decreased sedentary behaviour [28]. Additionally, the intervention effects on movement behaviour changes may have caused a risk for indirectness. In 1 study the intervention significantly decreased sedentary behaviour and increased LPA, but had no effect on MVPA [28]. In the other study [27], the intervention had no effect on sedentary time and TPA, while the control group showed improvements in MVPA

^d^ Quality of evidence was downgraded from “high” to “low” due to serious risk of bias and serious indirectness

^e^ Includes 1 non-randomized intervention [29]

^f^ Serious risk of bias. No control group

^g^Quality of evidence was downgraded from “low” to “very low” due to serious risk of bias

^h^Includes 2 longitudinal studies [30, 36]

^i^ Serious risk of bias. Both studies used convenience sampling for recruitment. One study measured movement behaviours via questionnaire and showed no evidence of psychometric testing; additionally, the analyzed sample (*n* = 915) and the full recruitment cohort (*n* = 2128) differed on parental ethnicity, education, and household income [36]

^j^ Serious indirectness. In 1 study, the method of classifying “less active” and “more active” groups resulted in groupings that did not significantly differ on sedentary time, but did differ on various components of LPA and MVPA (i.e., bouts per day, average minutes per bout, average MET score per bout, and total minutes per day) [30]

^k^ Dose-response & large magnitude of an effect. One study [36] showed evidence of dose-response where the group with high levels of sleep and low levels of TV time saw the most benefits for adiposity; as well, this study had a large magnitude of effect (i.e., compared to high sleep and low TV group, low sleep and high TV group had increased odds of overweight status (OR = 5.93; 95% CI = 2.03, 17.30))

^l^ Quality of evidence was downgraded from “low” to “very low” due to serious risk of bias and serious risk of indirectness; because of this limitation, was not upgraded for dose-response and large magnitude of an effect

^m^ Includes 3 cross-sectional studies [33–35]

^n^ Serious risk of bias. All studies used subjective measurements (questionnaires) with inadequate consideration of psychometric testing. One study used convenience sampling [33]

^o^ Quality of evidence was downgraded from “low” to “very low” due to serious risk of bias

For the experimental studies, two studies were clustered RCTs [27, 28], while one study was a non-randomized intervention [29]. Within both clustered RCTs, the interventions were designed to increase physical activity (structured physical activity program) and decrease sedentary behaviour (informational handouts) among preschool-aged children, primarily in child care settings. The control groups received usual care. Greater decreases in at least one measure of adiposity were observed in the intervention group compared to the control group in 1/2 studies, and no group differences were observed in 1/2 studies. Specifically, in one study, BMI z-scores were not significantly different between intervention and control groups at 6- and 12-month follow-up [27]. Significant differences between the intervention and control groups were also not observed for accelerometer-measured sedentary time and LPA. However, significant differences between groups were observed for MVPA at 6-month follow-up, as the control group was found to have higher MVPA [27]. In the other clustered RCT, body fat percentage and fat mass measured via bio-electrical impedance significantly decreased in the intervention group compared to the control group at 6-month follow-up, while no significant differences between groups were observed for BMI, BMI z-score, and fat-free mass [28]. In contrast to the first clustered RCT [27], significant increases in accelerometer-derived total PA and LPA as well as significant decreases in accelerometer-derived sedentary time were observed in the intervention group compared to the control group; however, no significant differences between groups were found for MVPA [28]. The quality of evidence was downgraded from “high” to “low” due to a serious risk of bias and serious indirectness (see Table 1).

The one non-randomized intervention involved provincial legislation targeting increased physical activity and decreased sedentary behaviour among toddlers and preschool-aged children within the child care setting [29]. A significant decrease in BMI z-score between baseline and 6-month follow-up was observed in toddlers but not preschool-aged children. However, the toddler age group showed significant decreases in sedentary time and increases in physical activity (MVPA but not LPA) between baseline and follow-up, whereas the preschool-aged group was found to have significant increases in sedentary time and decreases in physical activity (LPA but not MVPA) between baseline and follow-up. The quality of evidence was downgraded from “low” to “very low” due to a serious risk of bias (see Table 1).

For the observational studies, two studies were longitudinal [30, 36] and three studies were cross-sectional [33–35]. Within the longitudinal studies, one study assessed the combination of sedentary behaviour and physical activity among toddlers and preschool-aged children-aged children [30], while the other study assessed the combination of sleep and sedentary behaviour, specifically television (TV) viewing, among infants and toddlers [36]. Favourable associations between the most ideal combinations of movement behaviours and adiposity were observed in the study that included a combination of sleep and sedentary behaviour [36], whereas null associations were observed in the study that included the combination of sedentary behaviour and physical activity [30]. For the study that included sleep and sedentary behaviour [36], groups were made based on the average levels of sleep (i.e., high sleep ≥12 h, low sleep <12 h) and TV time (i.e., high TV ≥2 h, low TV <2 h) at ages 6 months, 1 year, and 2 years. When comparisons were made to the most ideal group (i.e., high sleep and low TV), the high sleep and high TV group did not differ on any of the 4 adiposity indicators at age 3; the low sleep and low TV group was unfavourably associated with 1/4 adiposity indicators at age 3; and the “least ideal” group (i.e., low sleep and high TV) was unfavourably associated with 3/4 adiposity indicators at age 3. For the study that included sedentary behaviour and physical activity, “active” and “less active” groups were created at six time-points over 2 years using Hidden Markov Modelling with accelerometer variables (i.e., total minutes of sedentary time per day, total minutes of LPA per day, total minutes of MVPA per day, average minutes per bout of MVPA, average metabolic equivalent [MET] score per MVPA bout, total bouts of MVPA per day, standard deviation of total MVPA bouts per day, and standard deviation of average MET score per MVPA bout) [30]. When comparing “active” and “less active” groups, no significant longitudinal differences were found for BMI percentile and weight percentile. Of note, all accelerometer variables were significantly different between the two groups, except time spent sedentary. The quality of evidence was downgraded from “low” to “very low” due to a serious risk of bias and serious indirectness (see Table 1).

The three cross-sectional studies examined the associations between the combinations of sedentary behaviour (specifically parent-reported screen time or TV viewing) and physical activity with adiposity [33–35]. The most ideal combinations of sedentary behaviour and physical activity were favourably associated with adiposity for at least one association in 1/3 studies, and not associated with adiposity in 2/3 studies. More specifically, an unfavourable association between a high ratio of screen time to physical activity and overweight classification was reported in one study among toddlers and preschool-aged children [33], while null associations were reported in the other two studies that stratified groups of preschool-aged children by levels and frequencies of screen time or TV viewing and physical activity [34, 35]. The quality of evidence was downgraded from “low” to “very low” due to a serious risk of bias (see Table 1).

#### Motor development

The association between combinations of sedentary behaviour and physical activity, and motor development were examined in two experimental studies (see Table 2 and Additional File 2: Table S2) [27, 31]. Assessments for motor development included the Movement Assessment Battery (composite of jumping, balance, skipping, and ball exercises) [27] and the Test of Gross Motor Development–2 (composite of locomotor and object control skills) [31]. Both studies were clustered randomized controlled trials primarily in child care settings, where the aim was to increase physical activity (structured physical activity program) and decrease sedentary behaviour (informational handouts) among preschool-aged children. In both studies, the control group received usual care. Greater increases in at least one motor development outcome in the intervention group compared to the control group were observed in both studies. Specifically, in one study, significant improvements in fundamental movement skills were observed in the intervention group compared to the control group at 6-month follow-up [25]. In the second study, significant improvements in scores for gross motor quotient and locomotor skills were observed in the intervention group compared to the control group at 6-month follow-up, although no significant group differences were observed for object control skills [29]. However, as described above in the results for adiposity, in one study, sedentary time and LPA did not significantly differ between groups, and MVPA was significantly higher in the control group at 6-month follow-up [25]. The quality of evidence was downgraded from “high” to “low” due to a serious risk of bias and serious indirectness (see Table 2).

**Table 2:** The relationship between movement behaviours and motor development

No. of studies	Design	Quality assessment					No. of participants	Absolute effect	Quality
		Risk of bias	Inconsistency	Indirectness	Imprecision	Other			
Mean baseline ages were 3.3 and 4.2 years. Data were collected by clustered RCT (*n* = 2). Motor development was assessed via the Movement Assessment Battery for Children and the Test of Gross Motor Development–2.									
2	Cluster RCT^a^	Serious risk of bias^b^	No serious inconsistency	Serious indirectness^c^	No serious imprecision	None	1245	SB + PA: The movement behaviour interventions were *favourably* associated with overall motor skills in 2 studies [27, 31].	LOW^d^

*LPA* light-intensity physical activity, *MVPA* moderate- to vigorous-intensity activity, *RCT* randomized controlled trial, *TPA* total physical activity

^a^ Includes 2 cluster RCTs [27, 31]

^b^ Serious risk of bias. In 1 study sex was not adjusted for in the analysis [31]

^c^ Serious indirectness. The sedentary behaviour components of the interventions were minimal in both interventions, which could have caused a risk for indirectness. However, significant reductions in sedentary time were observed in 1 study [31]. Additionally, the intervention effects may have caused a risk for indirectness. In 1 study the intervention significantly decreased sedentary behaviour and increased LPA, but had no effect on MVPA [31]. In the other study [27], the intervention had no effect on sedentary time and TPA, while the control group showed improvements in MVPA

^d^ Quality of evidence was downgraded from “high” to “low” due to serious risk of bias and serious indirectness

#### Fitness

The association between combinations of sedentary behaviour and physical activity, and fitness were assessed in one observational study among preschool-aged children (see Table 3 and Additional File 2: Table S3) [32]. Fitness was assessed using the PREFIT fitness test battery, consisting of the 20-m shuttle run, handgrip strength, standing long jump, and the 4 × 10 shuttle run. This cross-sectional study used a predictive model (i.e., isotemporal substitution) for the analysis. Replacing sedentary time with LPA was unfavourably associated with 1/4 fitness indicators, while replacing sedentary time with vigorous-intensity physical activity (VPA) was favourably associated with 3/4 fitness indicators. Of note, replacing sedentary time with moderate-intensity physical activity (MPA) was not associated with any fitness indicators. The quality of evidence was downgraded from “low” to “very low” due to a serious risk of bias (see Table 3).

**Table 3:** The relationship between movement behaviours and fitness

No. of studies	Design	Quality assessment					No. of participants	Absolute effect	Quality
		Risk of bias	Inconsistency	Indirectness	Imprecision	Other			
Mean baseline age of 4.48 years. Data were collected by cross-sectional (*n* = 1) study design. Fitness was assessed using the PREFIT fitness test battery, and included cardiorespiratory fitness (i.e., 20-m shuttle run), muscular fitness (i.e., handgrip strength and standing long jump), and speed-agility (i.e., 4 × 10-m shuttle run).									
1	Cross-sectional^a^	Serious risk of bias^b^	No serious inconsistency	No serious indirectness	No serious imprecision	Exposure/ indicator gradient^c^	307	SB + PA: Replacing SB with LPA was unfavourably associated with standing long jump, and not associated with 20-m shuttle performance, handgrip strength, or 4 × 10-m shuttle performance. Replacing SB with MPA was not associated with 20-m shuttle performance, handgrip strength, standing long jump, or 4 × 10-m shuttle performance. Replacing SB with VPA was favourably associated with 20-m shuttle performance, standing long jump, and 4 × 10-m shuttle performance, and not associated with handgrip strength.	VERY LOW^d^

*LPA* light-intensity physical activity, *MPA* moderate-intensity physical activity, *MVPA* moderate- to vigorous-intensity activity, *PA* physical activity, *SB* sedentary behaviour, *TPA* total physical activity, *VPA* vigorous-intensity physical activity

^a^ Includes 1 cross-sectional study [32]

^b^ Serious risk of bias. This study used convenience sampling for recruitment. As well, the analysis relied on predictive modelling (i.e., isotemporal substitution) instead of explanatory modelling (e.g., linear regression)

^c^ Exposure/indicator gradient. A gradient for higher TPA, MVPA, VPA with higher fitness was observed

^d^ Quality of evidence was downgraded from “low” to “very low” due to serious risk of bias; because of this limitation, was not upgraded for an exposure/indicator gradient

#### Growth

The association between combinations of sedentary behaviour and physical activity, and growth were assessed in two studies (see Table 4 and Additional File 2: Table S4) [28, 30]. Growth was assessed with objective measures of height and weight [28], and weight percentile [30]. One study had an experimental design [28]; the other was observational [30].

**Table 4:** The relationship between movement behaviours and growth

No. of studies	Design	Quality assessment					No. of participants	Absolute effect	Quality
		Risk of bias	Inconsistency	Indirectness	Imprecision	Other			
Mean baseline age of 3.3 years, and range of 2.5-3.5 years. Data were collected by cluster RCT (*n* = 1) and longitudinal study design (*n* = 1). Height and weight were objectively measured in both studies.									
1	Cluster RCT^a^	Serious risk of bias^b^	No serious inconsistency	No serious indirectness^c^	No serious imprecision	None	83	SB + PA: The movement behaviour intervention was *not* associated with changes in height or weight [28].	MODERATE^d^
1	Longitudinal^e^	No serious risk of bias^f^	No serious inconsistency	Serious indirectness^g^	No serious imprecision	None	248	SB + PA: Classification based on accelerometer variables did *not* predict weight percentile over 2 years [30].	VERY LOW^h^

*LPA* light-intensity physical activity, *MET* metabolic equivalent, *MVPA* moderate- to vigorous-intensity activity, *RCT* randomized controlled trial, *TPA* total physical activity

^a^ Includes 1 cluster RCT [28]

^b^ Serious risk of bias. Age was not adjusted for in the analysis

^c^ No serious indirectness. The sedentary behaviour component of the intervention was minimal, which could have caused a risk for indirectness. However, the intervention did lead to significantly reduced sedentary time [28]. Additionally, while the intervention had no effect on MVPA, it did lead to increased TPA and LPA

^d^ Quality of evidence was downgraded from “high” to “moderate” due to serious risk of bias

^e^ Includes 1 longitudinal study [30]

^f^ No serious risk of bias. This sample was recruited using convenience recruiting

^g^ Serious indirectness. The method of classifying “less active” and “more active” groups did not create groups that significantly differed on sedentary time, but did differ on various components of LPA and MVPA (i.e., bouts per day, average minutes per bout, average MET score per bout, and total minutes per day)

^h^ Quality of evidence was downgraded from “low” to “very low” due to serious indirectness

The experimental study was a clustered RCT [28]. Significant differences between the intervention group (structured physical activity program, and informational hand-outs for sedentary behaviour) and the control group (usual care) for height and weight were not observed at 6-month follow-up. The quality of evidence was downgraded from “high” to “moderate” due to a serious risk of bias (see Table 4).

The observational study was a longitudinal study where “active” and “less active” groups were created at six time-points over 2 years [30]. No significant longitudinal differences in weight percentile were found when comparing “active” and “less active” groups [30]. However, as mentioned above in the results for adiposity, significant group differences were observed for physical activity variables but not for sedentary time in this study. The quality of evidence was downgraded from “low” to “very low” due to a serious risk of bias and serious indirectness (see Table 4).

## Discussion

The purpose of this systematic review was to determine the relationships between combinations of movement behaviours and health indicators in early years children (aged 1.00 month to 4.99 years). Ten studies, published over the last 10 years (2006-2016), were included that assessed the relationships between the combinations of sedentary behaviour and physical activity, or sleep and sedentary behaviour, with the indicators of adiposity, motor development, fitness, and growth. Across observational and experimental study designs, the most ideal combinations of sedentary behaviour and physical activity (e.g., low sedentary behaviour and high physical activity) were favourably associated with motor development (2 RCTs), and fitness (1 cross-sectional study) among preschool-aged children; both favourably (1 RCT, 1 non-randomized intervention, and 1 cross-sectional study) and not associated (1 RCT, 1 longitudinal study, and 2 cross-sectional studies) with adiposity among toddlers and preschool-aged children; and not associated with growth (1 RCT and 1 longitudinal) among toddlers and preschool-aged children. Furthermore, the most ideal combinations of sleep and sedentary behaviour (e.g., high sleep and low sedentary behaviour) were favourably associated with adiposity (1 longitudinal study) among infants and toddlers. The quality of the evidence ranged from “very low” to “moderate”, with most ratings being “very low” or “low”, and all evidence being downgraded at least once. Table 5 presents a summary of the findings.

**Table 5:** Summary of overall findings

Health indicator	No. of studies	No. of participants	Quality of evidence	Summary of findings
Critical				
Adiposity	8	7046	Very low to low	3/7 studies that examined the most ideal combinations of sedentary behaviour and physical activity found favourable associations with at least 1 adiposity indicator. 1/1 study that examined the most ideal combinations of sleep and sedentary behaviour found favourable associations with at least 1 adiposity indicator.
Motor development	2	1245	Low	2/2 studies that examined the most ideal combinations of sedentary behaviour and physical activity found favourable associations with at least 1 motor development indicator.
Fitness	1	307	Very low	1/1 study that examined the most ideal combinations of sedentary behaviour and physical activity found favourable associations with at least 1 fitness indicator.
Growth	2	331	Very low to moderate	No studies that examined the most ideal combinations of sedentary behaviour and physical activity found favourable associations with at least 1 growth indicator.

Within the current review, some parallels were seen with a similar review on school-aged children and youth [10]. Specifically, both reviews were based on limited evidence produced in the last 10 years, with sedentary behaviour and physical activity combinations being investigated in the majority of studies. Additionally, within both reviews, all studies examining the most ideal combinations of sedentary behaviour and physical activity (e.g., low sedentary behaviour and high physical activity) found favourable associations with fitness, although it should be noted that only one study in the current review assessed fitness, compared to three studies in the school-aged children and youth review.

Another parallel between the current review and the school-aged children and youth review [10] was the lack of intermediate movement behaviour combinations examined. Specifically, the majority of research in both reviews compared the most ideal combinations (e.g., high levels of physical activity and low levels of screen time) to the least ideal combinations (e.g., low levels of physical activity and high levels of screen time), with no consideration of the intermediate combinations (e.g., high levels of physical activity and high levels of screen time). Within the current review, only one study looked at intermediate combinations of movement behaviours [36]. More specifically, Taveras and colleagues created four groups that could be defined as: (1) “most ideal” (i.e., high sleep and low TV time), (2) “poor TV time” (i.e., high sleep and high TV time), (3) “poor sleep” (i.e., low sleep and low TV time) and (4) “least ideal” (i.e., low sleep and high TV time). When compared to the “most ideal” group (reference group), no associations with adiposity measures were found in the “poor TV time” group; one unfavourable association was observed with adiposity measures in the “poor sleep” group; and three unfavourable associations with adiposity measures were reported for the “least ideal” group. Furthermore, a very large magnitude of effect was found only when comparing the “least ideal” and “most ideal” groups. Taken together, this reinforces the importance of studying the combinations of ideal movement behaviours for health indicators (rather than studying movement behaviours in isolation), and this emphasis could be incorporated into future guidelines. However, this conclusion should be interpreted with caution, since it is based on only one study in this age group. Further research is needed to determine the robustness of this conclusion.

While adiposity was the most investigated health indicator in this review as well as the school-aged children and youth review [10], the trends in the associations between combinations of physical activity and sedentary behaviour, and adiposity, were inconsistent between the two reviews. Specifically, for the current review the most ideal combinations of sedentary behaviour and physical activity (e.g., low sedentary behaviour and high physical activity) were favourably associated with adiposity in 3/7 studies but showed null associations in the remaining 4/7 studies. However, for the school-aged children and youth review, the most ideal combinations of sedentary behaviour and physical activity were favourably associated with adiposity in 8/8 studies [10]. A possible explanation for this difference in age groups is the accumulative nature of adiposity, as it has been observed that the prevalence of overweight and obesity increases as children progress from the early years to adolescence [37]. Additionally, weight-for-height trajectories typically increase during infancy, decrease to the lowest point in the preschool years, then rebound to increase into adulthood [38–40]. Considering there is variability in the ages at which these shifts occur during the early years (e.g., peak at 9-12 months, rebound at 3-7 years), determining the association between movement behaviours and adiposity markers is more complicated in the early years age group.

Though a number of comparisons can be made between the current review and the school-aged children and youth review [10], it was not possible to compare the associations between combinations of sleep and sedentary behaviour with adiposity across the reviews, given that only one study was included in each review. Additionally, it was not possible to compare findings between reviews for the health indicators motor development and growth, since within the school-aged and youth review [10], growth was not a relevant indicator and no studies were found for motor development. Clearly, there is a need for future research on the associations between other combinations of movement behaviours (e.g., sleep, sedentary behaviour, and physical activity) and a variety of health indicators within the pediatric population.

One potential reason for the lack of evidence on the combinations of all movement behaviours and health indicators in early years children, and in other age groups, is the challenge of measuring the full spectrum of movement behaviours in the field. Specifically, valid and reliable measurements are needed for all the movement behaviours in the sleep-wake cycle (e.g., daytime and nighttime sleep, sedentary time, LPA, and MVPA in a 24-h period). Ideally these behaviours would be captured with one tool, as some feel the relatively small increases in validity from wearing multiple monitors do not justify the increased participant burden and research costs [41]. The feasibility of such a task has been demonstrated in older children, as 24-h protocols have proven to increase accelerometer wear-time compliance [42]. However, the validity of measuring all behaviours with one tool remains unknown/untested in this age group, though it has been demonstrated that objectively measuring sleep, sedentary time, and physical activity with one device does not currently produce valid estimates for all behaviours in adults [43].

Another related measurement issue specific to this review is that current objective measures of sedentary behaviour capture total sedentary time but not the time spent in specific types of sedentary behaviour (e.g., TV viewing). However, in isolation, specific types of sedentary behaviour, such as screen time and TV viewing, have shown more consistent relationships with health indicators compared to objectively measured sedentary time in pediatric populations [21, 44]. Interestingly, within the current review, when sedentary behaviour was included as part of a combination, similar findings were observed regardless of whether the exposure was screen time, TV viewing, or objectively measured sedentary time. Given that a limited number of studies were included in this review, further research that simultaneously measures screen time and non-screen-based sedentary time is required to better understand the health implications of different combinations of sedentary behaviour and other movement behaviours.

The challenge of measuring the full spectrum of movement behaviours is further complicated in early years children. For instance, most sleep research occurring in free-living conditions for early years children has relied on accelerometry as a valid and reliable objective measure [45]. However, to date one of the biggest gaps in accelerometry-derived sleep research is the lack of a valid and reliable method to distinguish daytime sleep (i.e., napping) from behaviours sharing similar accelerometer characteristics (e.g., non-wear time, long bouts of sedentary time) [46]. Additionally, there may be variability in how the full spectrum of movement behaviours is measured in infants who are non-ambulatory compared to older age groups. For example, an accelerometer would have difficulties determining tummy time in infants. Additionally, the movements recorded from an accelerometer in infants may represent the movements of a parent carrying a child [47]. Therefore, current estimates of the full spectrum of movement behaviours occurring during a sleep-wake cycle must either rely on methods with suboptimal validity and reliability (e.g., questionnaires, removing naps with non-wear time protocols) or incorporate multiple measurement tools, thereby increasing participant and researcher burden.

Once movement behaviours have been measured, another challenge exists in the analysis of these data. To date, most of the research involving multiple movement behaviours has used multivariate analyses attempting to determine the independent effects of a movement behaviour with a health indicator, while mutually adjusting for other movement behaviours [48]. For instance, within this supplemental issue, approximately 10% of studies in the sedentary behaviour review had analyses that adjusted for physical activity [21]. Furthermore, in a similar review in school-aged children and youth, this mutual adjustment for physical activity was observed in more than half of the included studies [44]. Debate exists regarding the appropriateness of these mutual adjustments. Opponents would argue that within a 24-h period, altering one movement behaviour results in altering another movement behaviour (e.g., 1 h less sleep could result in 1 h more sedentary time); thus, movement behaviours are codependent and require analyses that are sensitive to this collinearity [48]. Addressing this topic, a recent review highlighted the current best practice techniques for analyzing movement behaviours [49]. Two examples of analyses included in the review that have been proposed to account for the integrated nature of movement behaviours were isotemporal substitution and compositional analyses [49]. However, the novelty of these techniques is reflected in the limited, albeit growing, evidence in the literature.

Within the current review, no studies were found using compositional analyses. However, within this supplemental issue, Carson et al. [2] employed compositional analyses to examine the integrated nature of movement behaviours, and their relationship with adiposity indicators in a nationally representative sample of Canadian preschool-aged children. This study found null relationships for sleep, sedentary time, LPA, and MVPA relative to one another; however, the overall composition of movement behaviours was favourably associated with BMI z-scores. Some researchers postulate that compositional analyses provide the most appropriate analytical solution for understanding the relationships between health indicators and movement behaviours over a 24-h period (i.e., a finite geometry) [49]. Future research should use compositional analyses to examine a diversity of health indicators in infants, toddlers, and preschool-aged children.

Examining the health implications of replacing movement behaviours with each other provides further insight into the importance of movement behaviour combinations. In the current review, one study by Leppanen and colleagues was included using isotemporal substitution analysis [32]. More specifically, within a preschool-aged sample, replacing sedentary time with VPA provided the most benefits for fitness indicators compared to MPA and LPA [32]. The isotemporal substitution model used by Leppanen et al. also found null relationships between replacing SB with LPA, MPA, and VPA on BMI and objectively measured fat mass [32]. However, a significant relationship was found between replacing sedentary time with VPA and fat free mass index. As well, Saunders and colleagues included two studies using isotemporal substitution models in the school-aged children and youth review [10]. Within these studies, favourable associations with adiposity [50, 51] and fitness [51] were observed when replacing sedentary time with MVPA, but not LPA [10]. The results of these three isotemporal analysis studies add important context regarding the potential health implications of not only increasing the amount of higher-intensity physical activity or decreasing sedentary time, but also of replacing sedentary time with higher-intensity physical activity across different age groups. Future guidelines therefore could highlight the importance of replacing sedentary time with higher-intensity physical activity in the early years. However, given that only one study in this review looked at movement behaviour replacement in a preschool-aged sample, researchers should continue to examine isotemporal substitutions of movement behaviours on a variety of health indicators in infants, toddlers, and preschool-aged children.

The adiposity results of Leppanen and colleagues’ isotemporal substitution analysis [32] were not presented in the results of this review due to an exclusion criteria for predictive models (see “Methods” section). The reason for this decision was that predictive models have less certain conclusions. More specifically, predictive models use a set of assumptions to make hypothetical inferences about the data, rather than direct evidence (e.g., linear regression), which uses actual measured data to make conclusions. However, as this field of research advances, it is becoming apparent that predictive models (e.g., isotemporal substitution and compositional substitution) can provide useful context when examining the relationships that combinations of movement behaviours have with one another and with health indicators. Determining the nuances of each statistical approach will be advantageous moving forward, as researchers will be able to select the best approach to accommodate their unique dataset and research questions.

Although some study findings with predictive modelling were excluded, studies with unbalanced combinations of movement behaviours, where the focus was primarily on one movement behaviour, were not excluded from the current review. For example, in two experimental studies, unbalanced targeting of combinations of movement behaviours may have occurred when the focus of the interventions was on structured physical activity but informational material on sedentary behaviour was also distributed. As well, in one observational study, an unbalanced combination of movement behaviours may have been examined when groups were created based on several physical activity variables and one sedentary time variable, but groups did not differ on the sedentary time variable. While including these studies enabled screening decisions to be objective and ensured all available evidence was captured, the evidence from these studies may be more heavily weighted on isolated movement behaviours compared to combinations of movement behaviours. Therefore, to truly understand the impact of the combination of movement behaviours, future studies that use more balanced approaches to intervene on various movement behaviours in the early years are needed.

The main strengths of this review were the rigorous methods employed to conduct the systematic review. Specifically, this review was prospectively registered in PROSPERO, comprehensive search strategies were developed and peer-reviewed by librarians with systematic review expertise, articles were screened with the DistillerSR Software, and quality of evidence was assessed using the GRADE framework. A limitation within this review was the decision to only include predictive modelling in the absence of evidence for a particular indicator of interest, since these types of analyses may more appropriately handle codependent data. However, only one study in this review used predictive modelling, of which the adiposity components were omitted but the fitness indicators were extracted and presented. Additionally, the inclusion of studies with potential unbalanced combinations of movement behaviours was another limitation, given that findings from this study were likely disproportionally weighted on a single movement behaviour (e.g., physical activity). However, this decision maximized the limited evidence base and minimized subjective inclusion criteria.

## Conclusions

In this review, across observational and experimental study designs, the most ideal combinations of sedentary behaviour and physical activity were favourably associated with motor development and fitness among preschool-aged samples; both favourably and not associated with adiposity among toddlers and preschool-aged children; and not associated with growth among toddlers and preschool-aged children. Additionally, replacing sedentary time with VPA was found to be beneficial for fitness among preschool-aged children. The most ideal combinations of sleep and sedentary behaviour were also found to be favourably associated with adiposity among infants and toddlers, especially when compared to groups of children with low levels of sleep and high levels of sedentary behaviour. Findings from this review can help inform the Evidence-to-Decision framework for creating guidelines that seek to promote ideal 24-h movement behaviours for optimal health and development in early years children. However, these conclusions are based on limited and primarily “very low” to “low” quality evidence, of which there was limited diversity of combinations of movement behaviours, health indicators, and age groups. Future high-quality studies should focus on including more diverse combinations of movement behaviours (e.g., sleep, sedentary behaviour, and physical activity), including intermediate combinations and a variety of health indicators across infant, toddler, and preschool-aged children age groups. Additionally, future studies should use appropriate methods for analyzing integrated combinations of movement behaviours.

## Additional files

Additional File 1: Search Strategies. (DOCX 19 kb)

Additional File 2: Table S1. Summary of included studies for adiposity. **Table S2.** Summary of included studies for motor development. **Table S3.** Summary of included studies for fitness. **Table S4.** Summary of included studies for growth. (DOCX 40 kb)

## Abbreviations

BMI: Body mass index
GRADE: Grading of Recommendations Assessment, Development and Evaluation
LPA: Light-intensity physical activity
MET: Metabolic equivalent
MPA: Moderate-intensity physical activity
MVPA: Moderate- to vigorous-intensity physical activity
PA: Physical activity
PICO: Population, Intervention, Comparator and Outcome
PRISMA: Preferred Reporting Items for Systematic Reviews and Meta-Analyses
RCT: Randomized controlled trial
SB: Sedentary behaviour
TV: Television
VPA: Vigorous-intensity physical activity
