# Supplementary material for: Systematic review of the relationships between combinations of movement behaviours and health indicators in the early years (0-4 years)
Source: BMC Public Health. 2017 Nov 20;17(Suppl 5):849. doi: 10.1186/s12889-017-4851-1 (PMC5773877; doi:10.1186/s12889-017-4851-1)
Supplement: Supplementary file 1 — Search Strategies. (DOCX 19 kb) [file 12889_2017_4851_MOESM1_ESM.docx]

**Additional file 1:** Search Strategies

Epub Ahead of Print, In-Process & Other Non-Indexed Citations, Ovid MEDLINE(R) Daily and Ovid MEDLINE(R) (1946 to November 1, 2016)

| **MEDLINE** |
| --- |
| 1. Physical Activity.mp. |
| 2. exp Exercise/ |
| 3. exp Exercise Movement Techniques/ |
| 4. exp Exercise Therapy/ |
| 5. Physical Exertion/ |
| 6. exp "Physical Education and Training"/ |
| 7. exp Sports/ |
| 8. (sport$ or bicycl$ or swim$ or walk$ or run$ or jog$).tw,kf. |
| 9. (physical$ adj2 activ$).tw,kf. |
| 10. (aerobic adj2 (train$ or active$)).tw,kf. |
| 11. "Play and Playthings"/ and (activ* or outdoor*).tw,kf. |
| 12. ((activ* or outdoor*) adj3 play*).tw,kf. |
| 13. playground*.tw,kf. |
| 14. active.ti. and (space* or behavio?r* or transport* or commut* or neighbo?rhood* or park* or game* or gaming or lifestyle).mp. |
| 15. (active adj3 (space* or behavio?r* or transport* or commut* or neighbo?rhood* or park* or game* or gaming or lifestyle)).tw,kf. |
| 16. prone position*.mp. or floor time.tw,kf. |
| 17. ((abdomen or stomach or tummy or belly) adj2 (time or play)).tw,kf. |
| 18. or/1-17 |
| 19. Sedentary Lifestyle/ |
| 20. (sedentary or inactiv* or (lack adj2 activity)).tw,kw. |
| 21. ((low adj3 energy expend*) or physical* inactiv*).tw,kw. |
| 22. ((chair or stroller or car or automobile* or auto or motor vehicle* or bus or indoor* or in-door or computer) adj3 time).tw,kw. |
| 23. sitting.tw,kw. |
| 24. Television/ or Video Games/ or Software/ or Videodisc Recording/ or Cartoons as Topic/ or Motion Pictures as Topic/ |
| 25. exp Internet/ or exp Computers, handheld/ |
| 26. Communications Media/ or Mass Media/ |
| 27. (television or screentime or ((screen or computer) adj3 time) or ((watch* or view*) adj2 (dvd* or video*)) or screen media or social media or video gam* or videogam* or computer gam* or electronic gam*).tw,kw. |
| 28. (Smartphone* or ipad or apps or app or mobile applications).tw,kw. |
| 29. screen based entertainment.tw,kw. |
| 30. or/19-29 |
| 31. Sleep/ |
| 32. (sleep adj3 duration).tw. |
| 33. exp *Sleep Apnea Syndromes/ and (apnea or apnoea).ti. |
| 34. (31 or 32) not 33 |
| 35. (18 and 30) or (18 and 34) or (30 and 34) |
| 36. limit 35 to ("infant (1 to 23 months)" or "preschool child (2 to 5 years)") |
| 37. 35 and (pre-school* or preschool* or early childhood).tw,kf. |
| 38. 36 or 37 |
| 39. limit 38 to (english or french) |
| 40. limit 39 to (journal article or published erratum or "retraction of publication") |
| 41. remove duplicates from 40 |

Ovid EMBASE (1980 to 2014 week 44)

| **EMBASE** |
| --- |
| 1. exp physical activity/ or exp exercise/ or exp kinesiotherapy/ or physical education/ or exp sport/ |
| 2. (sport* or bicycl* or swim* or walk* or run* or jog*).tw,kw. |
| 3. (aerobic adj2 (train$ or active$)).tw,kw. |
| 4. Play/ and (activ* or outdoor*).tw,kw. |
| 5. (((activ* or outdoor*) adj3 play*) or playground*).tw,kw. |
| 6. active.ti. and (space* or behavio?r* or transport* or commut* or neighbo?rhood* or park* or game* or gaming or lifestyle).mp. |
| 7. (active adj3 (space* or behavio?r* or transport* or commut* or neighbo?rhood* or park* or game* or gaming or lifestyle)).tw,kw. |
| 8. (prone position* or floor time).tw,kw. |
| 9. ((abdomen or stomach or tummy or belly) adj2 (time or play)).tw,kw. |
| 10. or/1-9 |
| 11. sedentary lifestyle/ |
| 12. (sedentary or inactiv* or (lack adj2 activity)).tw,kw. |
| 13. ((low adj3 energy expend*) or physical* inactiv*).tw,kw. |
| 14. ((chair or stroller or car or automobile* or auto or motor vehicle* or bus or indoor* or in-door or computer) adj3 time).tw,kw. |
| 15. sitting.tw,kw. |
| 16. bed rest.mp. |
| 17. television viewing/ or television/ or exp computer/ |
| 18. internet/ |
| 19. Social Media/ or Mobile Application/ or Mobile Phone/ |
| 20. (screen based entertainment or screen time).tw. |
| 21. (texting or text messag* or app or apps or mobile applications).tw. |
| 22. (smartphone* or smart phone* or cell phone* or mobile phone* or small screen*).tw. |
| 23. (iphone* or ipad* or ipod* or tablet* or laptop*).tw. |
| 24. (social media or Facebook or Youtube or Twitter or Snapchat or Instagram or Pinterest or Skype or Vine).tw. |
| 25. (television or screentime or ((screen or computer) adj3 time) or ((watch* or view*) adj2 (dvd* or video*)) or screen media or social media or video gam* or videogam* or computer gam* or electronic gam*).tw,kw. |
| 26. ((television adj watch*) or tv watch*).tw. |
| 27. or/11-26 |
| 28. Sleep/ |
| 29. Sleep Time/ |
| 30. (sleep adj3 duration).tw. |
| 31. exp Sleep Disordered Breathing/ and (apnea or apnoea).ti. |
| 32. (or/28-30) not 31 |
| 33. (10 and 27) or (10 and 32) or (27 and 32) |
| 34. limit 33 to (infant or preschool child <1 to 6 years>) |
| 35. 33 and (pre-school* or preschool* or early childhood).tw,kw. |
| 36. 34 or 35 |
| 37. limit 36 to conference abstract |
| 38. 36 not 37 |
| 39. limit 38 to (english or french) |
| 40. limit 39 to embase |
| 41. remove duplicates from 40 |

Ovid PsycINFO (1806 to October week 4 2016)

| **PsycINFO** |
| --- |
| 1. physical activity/ or exp exercise/ or activity level/ or movement therapy/ or dance therapy/ or mind body therapy/ or energy expenditure/ or physical education/ or exp sports/ |
| 2. (sport* or bicycl* or swim* or walk* or run* or jog*).tw,id. |
| 3. (physical* adj2 activ*).tw,id. |
| 4. (aerobic adj2 (train* or active*)).tw,id. |
| 5. (childhood play behavior/ or childhood play development/ or games/ or recreation/) and (activ* or outdoor*).tw,id. |
| 6. ((activ* or outdoor*) adj3 play*).tw,id. |
| 7. playgrounds/ or playground*.tw,id. |
| 8. active.ti. and (space* or behavio?r* or transport* or commut* or neighbo?rhood* or park* or game* or gaming or lifestyle).tw,id. |
| 9. (active adj3 (space* or behavio?r* or transport* or commut* or neighbo?rhood* or park* or game* or gaming or lifestyle)).tw,id. |
| 10. (prone position* or floor time).tw,id. |
| 11. ((abdomen or stomach or tummy or belly) adj2 (time or play)).tw,id. |
| 12. or/1-11 |
| 13. sedentar*.tw,id. |
| 14. ((low adj3 energy expend*) or physical* inactiv*).tw,id. |
| 15. ((chair or stroller or car or automobile* or auto or motor vehicle* or bus or indoor* or in-door or computer) adj3 time).tw,id. |
| 16. sitting.tw,id. |
| 17. ((television adj watch*) or tv watch* or cartoon*).tw,id. |
| 18. television viewing/ |
| 19. (television or screentime or ((screen or computer) adj3 time) or ((watch* or view*) adj2 (dvd* or video*)) or screen media or social media or video gam* or videogam* or computer gam* or electronic gam* or gaming).tw,id. |
| 20. screen based entertainment.tw,id. |
| 21. exp Social Media/ |
| 22. exp Mobile Devices/ |
| 23. (smartphone* or smart phone* or cell phone* or mobile phone* or small screen*).tw,id. |
| 24. (texting or text messag* or app or apps or mobile applications).tw,id. |
| 25. (iphone* or ipad* or ipod* or tablet* or laptop*).tw,id. |
| 26. bed rest.tw,id. |
| 27. or/13-26 |
| 28. Sleep/ or Sleep Deprivation/ |
| 29. (sleep adj3 duration).tw. |
| 30. *Sleep Apnea/ and (apnea or apnoea).ti. |
| 31. (28 or 29) not 30 |
| 32. (12 and 31) or (12 and 27) or (27 and 31) |
| 33. 32 and (pre-school* or preschool* or early childhood).mp. |
| 34. limit 32 to (140 infancy <2 to 23 mo> or 160 preschool age ) |
| 35. 33 or 34 |
| 36. limit 35 to (english or french) |
| 37. limit 36 to ("erratum/correction" or journal article) |
| 38. 37 or (36 and retraction.ti.) |
| 39. remove duplicates from 38 |
| EbscoHOST SportDiscus (1975 to Nov 1 2016)  **SportDiscus**  1. TI ( exercise or (physical* n2 activ*) OR aerobic N2 train* or aerobic N2 active* OR sport* or outdoor* OR playground or play or playing OR "rough and tumble" or "active recreation*" OR run* OR walk* OR jog* OR bicycl* or biking or cycling or tricylc* or "tummy time" or "floor time" or prone position or crawl* OR swim* or soccer or gymnastic* ) OR SU (exercise or (physical* n2 activ*) OR aerobic N2 train* or aerobic N2 active* OR sport* or outdoor* OR playground or play or playing OR "rough and tumble" or "active recreation*" OR run* OR walk* OR jog* OR bicycl* or biking or cycling or tricylc* or "tummy time" or "floor time" or prone position or crawl* OR swim* or soccer or gymnastic* ) |
| 2. Sleep |
| 3. DE "MASS media" or DE "INTERNET" OR DE "ELECTRONIC games" OR DE "INTERNET games" OR DE "MULTIPLAYER games" OR DE "VIDEO games") or DE "SEDENTARY behavior" or DE "SEDENTARY behavior in children" or (Smartphone* or ipad or apps or app or mobile applications) or screen based entertainment or (television N3 time or screentime or screen N3 time or computer N3 time or (watch* N2 dvd or watch N2 video* or view* N2 dvd* or view N2 video*)) or screen media or social media or video gam* or videogam* or computer gam* or electronic gam* OR chair N3 time or stroller N3 time or car N3 time or automobile* N3 time or auto N3 time or motor vehicle* N3 time or bus N3 time or indoor* N3 time or in-door N3 time or computer N3 time OR sitting or ((sedentary or inactiv* or (lack N2 activity))) or ((low N3 energy expend*) or physical* inactiv*) |
| 4. (S1 and S2) or (S1 and S3) or (S2 and S3) |
| 5. S4 and (Infan* or pre-school* or preschool* or early childhood or SU child*) |
