# Supplementary material for: Systematic review of the relationships between combinations of movement behaviours and health indicators in the early years (0-4 years)
Source: BMC Public Health. 2017 Nov 20;17(Suppl 5):849. doi: 10.1186/s12889-017-4851-1 (PMC5773877; doi:10.1186/s12889-017-4851-1)
Supplement: Supplementary file 2 — Summary of included studies for adiposity. Table S2. Summary of included studies for motor development. Table S3. Summary of included studies for fitness. Table S4. Summary of included studies for growth. (DOCX 40 kb) [file 12889_2017_4851_MOESM2_ESM.docx]

**Additional file 2**

**Table S1. Summary of included studies for adiposity**

| Refid #. Author (year); country | Study Design | Sample | Exposure | Indicator | Indicator Category | Main Findings |
| --- | --- | --- | --- | --- | --- | --- |
| 1246. Reilly et al. (2006) [27]. Scotland. | Clustered RCT | n=1162; mean age: 4.2 years | Intervention (6 months, 12 month follow-up):  **Sedentary behaviour:**  Received educational leaflet for families to reduce the time spent watching TV.  **Physical Activity:**  Nursery component was 3, 30 min sessions of PA each week over 24 weeks, delivered by staff who received three training sessions. For six weeks, posters also displayed focused on increasing PA through walking and play.  Home component consisted of families receiving resource packages with guidance on linking PA at nurseries and at home. As well, a physical activity educational leaflet.  Control:  Usual nursery curriculum, and agreement from head teachers not to enhance physical development and movement curriculum. | BMI calculated with objectively measured height and weight, and transformed into z-scores using the Cole criteria. | Adiposity | Intervention did not have an effect on BMI z-scores at post-intervention (6-months; p=0.87), or at follow-up (12-months; p=0.90).    *Note:* No intervention effects on accelerometer- derived sedentary time or TPA, but control group did have higher MVPA. |
| 26. Goldfield et al. (2016) [28]. Canada | Clustered RCT | n=83; mean age: 3.3 years | Intervention (6 months):  **Sedentary Behaviour:**  In addition to the PA component of intervention, parents were given the Canadian Society for Exercise Physiology Sedentary Behaviour Guidelines for the early years.  **Physical Activity**:  Two 3-hour training workshops to child care providers were delivered by an early learning PA specialist, a resource training manual, and some basic equipment (eg, balls, skipping ropes). Training binders included: physical activity guidelines, recommended weekly activity program, logsheet, as well as contingency plans for bad weather.  In addition there were 12, 1 hour "booster" sessions provided biweekly.  Control:  Wait list control continued to provide their standard childcare curriculum during the study period. | % body fat, fat mass (kg), and fat free mass (kg) assessed by hand and foot electrode-based bioelectrical impedance analysis.  BMI calculated by objective height and weight measurement, and transformed into z-BMI with WHO age- and sex- specific cutpoints. | Adiposity | Intervention was associated with decreased % body fat (Mean group x time difference= -1.9% ; 95%CI: -3.5, -0.3; p=0.023) and fat mass (Mean group x time difference= -0.3kg ; 95%CI: -0.7, -0.1; p=0.018).  However, no differences were found for fat free mass (Mean group x time difference= 0.2 kg; 95%CI: -0.07, 0.5; p=0.126), BMI (Mean group x time difference= -0.2 kg/m^2^; 95%CI: -0.1, 0.4; p=0.255), and BMI-z (Mean group x time difference= -0.12; 95%CI: -0.3, 0.07; p=0.225).  Note: Significant group x time interaction for TPA (Control: -3.1 min/day; intervention: 19.4 min/day), LPA (Control: -8.3 min/day; intervention: 7.8 min/day), and sedentary time (Control: 10.2 min/day; intervention: -12.3 min/day). No group x time interaction for MVPA (Control: 5.3 min/day; intervention: 11.8 min/day, p=0.085). |
| 2789. Carson et al. (2015) [29]. Canada | Non-Randomized Intervention | n=86; mean age: baseline: 39.5 months, follow-up: 46.3 months  Results stratified into toddlers and preschoolers | Intervention (6.5 months):  **Sedentary behaviour:**  Legislation regarding sedentary behaviour in accredited child care settings was released in the province of Alberta, which emphasized minimizing time children are sedentary. Centres going through accreditation were informed of these changes, and received multiple levels of support to create and achieve goals around sedentary behaviour.  **Physical activity:**  Legislation regarding physical activity in accredited child care settings was released in the province of Alberta, which emphasized promoting physical activity for children. Centres going through accreditation were informed of these changes, and received multiple levels of support to create and achieve goals around sedentary behaviour.  Control:  No control condition | Objectively measured height and weight used to calculate BMI, which was transformed into age- and sex-specific z-scores based on the WHO criteria. | Adiposity | Toddlers (19-35 months of age) showed a significant decrease in BMI z-scores (Baseline=0.62, SE=0.12; Follow-up=0.44, SE=0.10; P < 0.05), whereas preschoolers (36-60 months) showed no significant changes in BMI z-scores (Baseline=0.42, SE=0.17; Follow-up=0.36, SE=0.19; P > 0.05).  Note:  Toddlers showed significant decreases in sedentary time (Baseline=37.8 min/hr, SE=0.7; Follow-up=34.7 min/hr, SE=0.8; P < 0.05), and increases in MVPA (Baseline=4.0 min/hr, SE=0.4; Follow-up=5.7 min/hr, SE=0.4; P < 0.05), but no changes in LPA (Baseline=18.1 min/hr, SE=0.6; Follow-up=19.6 min/hr, SE=0.6; P > 0.05). Whereas preschoolers showed a significant increase in sedentary time (Baseline=35.2 min/hr, SE=1.1; Follow-up=37.1 min/hr, SE=0.5; P < 0.05), and decrease in LPA (Baseline=19.5 min/hr, SE=0.6; Follow-up=17.6 min/hr, SE=0.3; P < 0.05); but no changes in MVPA (Baseline=5.3 min/hr, SE=0.7; Follow-up=5.3 min/hr, SE=0.3; P > 0.05). |
| 1149. Taveras et al. (2008) [36]. USA | Longitudinal | n=1579; age at baseline: 6 months (no mean reported), final time point= 3 years (no mean reported) | **Sleep:**  Parent reported total sleep duration at ages 6 months, 1 year, and 2 years; used to created weighted average.  High sleep defined as ≥12 hours/day.  **Sedentary Behaviour:**  Parent reported TV/video watching duration at ages 6 months, 1 year, and 2 years; used to created weighted average. High TV viewing defined as ≥2 hours/day.  **Combination:**  Created combinations of groups based on high and low classifications for sleep and TV time (e.g., high sleep and low TV) | BMI calculated with objectively measure height and weight, converted to age- and sex-specific z-scores based on CDC cutoffs, also categorized as at risk for overweight (85^th^-95^th^ percentile), and overweight (≥95^th^ percentile).  Subscapular and tricep skinfold thickness also measured for sum of two sites and ratio of the two thicknesses (subscapular/tricep). | Adiposity | Compared to HIGH SLEEP and LOW TV group, HIGH SLEEP and HIGH TV group did not differ on BMI-z (β=0.19; 95%CI= -0.04, 0.41), sum of skinfold thickness (β= 0.27; 95%CI= -0.69, 1.24), skinfold thickness ratio (β= -0.01; 95%CI= -0.05, 0.03), and overweight status (OR=1.91; 95%CI=0.69, 5.28).  Compared to HIGH SLEEP and LOW TV group, LOW SLEEP and HIGH TV group had higher BMI-z (β=0.35; 95%CI= 0.09, 0.62), sum of skinfold thickness (β= 1.62; 95%CI= 0.47, 2.78), and overweight status (OR=5.93; 95%CI=2.03, 17.30); but did not differ on skinfold thickness ratio (β= 0.04; 95%CI= -0.01, 0.09).  Compared to HIGH SLEEP and LOW TV group, LOW SLEEP and LOW TV group had higher BMI-z (β=0.16; 95%CI= 0.01, 0.31); but did not differ on sum of skinfold thickness (β= 1.62; 95%CI= 0.47, 2.78), skinfold thickness ratio (β= 0.04; 95%CI= -0.01, 0.09), and overweight status (OR=1.83; 95%CI=0.87, 3.85). |
| 11. Ip et al. (2016). [30]. USA | Longitudinal | n=248; Age range: 2.5-3.5 years | **Sedentary Behaviour:**  Accelerometer-derived minutes/day sedentary time  **Physical Activity:**  average total minutes of  light activity per day, average minutes per bout of MVPA, average MET score per MVPA bout, total minutes of MVPA per day, total bouts of MVPA per day, standard deviation of total MVPA bouts per day, standard deviation of average MET score per MVPA bout.  **Combination:**  All of these variables used (as well as autocorrelation between observations) to create less active and more active groups using hidden Markov modeling. | BMI calculated by objective height and weight measurement, and transformed into CDC percentiles. | Adiposity | Belonging to the more active group was not associated with BMI percentile over the 2 year duration of the study (β=0.56; SE=1.70; p=0.74).  Note: More active state was associated with higher LPA (total bouts per day (p<0.01), average minutes per bout (p<0.001), average MET score per bout (p<0.001), and total minutes per day (p=0.01)), MVPA (total bouts per day (p<0.001), average minutes per bout (p<0.001), average MET score per bout (p<0.001), and total minutes per day (p<0.001)), but not with minutes per day sedentary (p=0.09) which was the only sedentary variable. |
| 812. Nelson et al. (2006) [33]. USA. | Cross-Sectional | n= 593; age range: 2-4 years | **Sedentary Behaviour:**  Parental survey adapted from NHANES “... television-viewing and computer use patterns...Medians were used to determine ...average screen time…"  **Physical Activity:**  “…how many minutes or hours per day their children spent playing actively or exercising…”  **Combination:**  Calculated a screen time:active play time ratio |  | Adiposity | Children with more than twice as much screen time:active play time were at increased odds off having a BMI ≥ 85th%ile, compared to children with twice as much or less screen time:Active play time (OR= 1.61; 95%CI: 1.04-2.48; p=0.04) |
| 1028. Manios et al. (2009) [34]. Greece. | Cross-Sectional | n=2518; mean age (41.5 months) | **Sedentary Behaviour:**  Parent reported TV viewing time (hours/day) categorized as <2 hours/day and ≥2 hours/day.  **Physical Activity:**  Parent reported activities which were considered of light- to vigorous-intensity (>4 METS) (hours/week), categorized as <3 hours/week and ≥3 hours/week.  **Combination:**  Sample was stratified into <3 hours/week LVPA and ≥3 hours/week LVPA, and then tested if TV viewing predicted adiposity. | BMI calculated with objectively measure height and weight, and categorized as obese using the CDC cutoffs. | Adiposity | TV time not associated with obesity classification (p=0.135) when group stratified by <3 hours/week LVPA and ≥3 hours/week LVPA, after controlling for total energy intake (model not adjusted for energy intake was significantly associated). |
| 1101. Anderson et al. (2008) [35]. USA. | Cross-Sectional | n=777; mean age: 59.6 months | **Sedentary Behaviour:**  Parent reported screen time (TV and computer time) hours/day (NHANES data). High screen time defined as >2 hours/day.  **Physical Activity:**  Parent reported times/week child played or exercised actively. Low active defined as < 7 times/week.  **Combination:**  Children grouped into both High screen time AND low active group. | BMI calculated with objectively measure height and weight, and categorized as obese using the CDC cutoffs. | Adiposity | Boys with low active and high screen time showed no difference in percent obese vs. non-obese.  Girls with low active and high screen time showed no difference in percent obese vs. non-obese. |

95%CI = 95% confidence interval; BMI = body mass index; CDC = Center for Disease Control; hr = hours; kg = kilogram; LPA = light-intensity physical activity; LVPA = light- to vigorous-intensity physical activity; MET = metabolic equivalent; min = minutes; MPA = moderate-intensity physical activity; MVPA = moderate- to vigorous-intensity physical activity; NHANES = National Health and Nutrition Examination Survey; OR = odds ratio; PA = physical activity; RCT = randomized controlled trial; SE = standard error; TPA = total physical activity; TV = television; WHO = World Health Organization.

**Table S2. Summary of included studies for motor development**

| Refid #. Author (year); country | Study Design | Sample | Exposure | Indicator | Indicator Category | Main Findings |
| --- | --- | --- | --- | --- | --- | --- |
| 1246. Reilly et al. (2006) [27]. Scotland. | Clustered RCT | n=1162; mean age: 4.2 yr | Intervention (6 months, 12 month follow-up):  **Sedentary behaviour:**  Received educational leaflet for families to reduce the time spent watching TV.  **Physical Activity:**  Nursery component was 3, 30 min sessions of PA each week over 24 weeks, delivered by staff who received three training sessions. For six weeks, posters also displayed focused on increasing PA through walking and play.  Home component consisted of families receiving resource packages with guidance on linking PA at nurseries and at home. As well, a physical activity educational leaflet.  Control:  Usual nursery curriculum, and agreement from headteachers not to enhance physical development and movement curriculum. | Movement assessment battery (The battery provides a global motor skills score of 0-15, which is a composite of performance in jumping, balance, skipping, and ball exercises.) | Motor Development | Intervention nurseries had higher fundamental movement skills post-intervention, compared to control (Intervention post-intervention= 11.5, SD = 2.3; control post-intervention= 10.7, SD = 2.5)  *Note:* No intervention effects on accelerometer- derived sedentary time or TPA, but control group did have higher MVPA. |
|  |  |  |  |  |  |  |
| 4486. Adamo et al. (2016) [31]. Canada | Clustered RCT | N = 83; mean age=3.3yr | Intervention (6 months):  **Sedentary Behaviour:**  In addition to the PA component of intervention, parents were given the Canadian Society for Exercise Physiology Sedentary Behaviour Guidelines for the early years.  **Physical Activity**:  Two 3-hour training workshops to child care providers were delivered by an early learning PA specialist, a resource training manual, and some basic equipment (eg, balls, skipping ropes). Training binders included: physical activity guidelines, recommended weekly activity program, logsheet, as well as contingency plans for bad weather.  In addition there were 12, 1 hour "booster" sessions provided biweekly.  Control:  Wait list control continued to provide their standard childcare curriculum during the study period. | Test of Gross Motor Development (TGMD-2), broken into locomotor skills and object control skills, additionally the composite score of locomotor skills and object control skills (Gross motor quotient, or GMQ) | Motor Development | The intervention group had significantly greater changes in GMQ (Mean group x time difference: 5.70; 95% CI: 0.74, 10.67; p=0.025), GMQ percentile (Mean group x time difference: 13.33; 95% CI: 2.17, 24.49; p=0.020), locomotor skills (Mean group x time difference: 1.20; 95% CI: 0.18, 2.22; p=0.022), locomotor skills percentile (Mean group x time difference: 12.59; 95% CI: 1.97, 23.20; p=0.021); but showed no difference for object control skills (Mean group x time difference: 0.56; 95% CI: -0.41, 1.53; p=0.252) and object control skills percentile (Mean group x time difference: 6.40; 95% CI: -4.59, 17.39; p=0.249)  Note: State intervention associated with improvements in TPA but not MVPA. Based on Goldfield et al. 2015, it can be assumed that intervention also significantly increased LPA and decreased sedentary time. |

95%CI = 95% confidence interval; LPA = light-intensity physical activity; min = minutes; MVPA = moderate- to vigorous-intensity physical activity; PA = physical activity; RCT = randomized controlled trial; SD = standard deviation; TPA = total physical activity; TV = television.

**Table S3. Summary of included studies for fitness**

| Refid #. Author (year); country | Study Design | Sample | Exposures | Indicator | Indicator Category | Main Findings |
| --- | --- | --- | --- | --- | --- | --- |
| 33. Leppänen et al. (2016) [32]. Sweden | Cross-sectional | n=307; mean age: 4.48 years | **Sedentary Behaviour**  Accelerometer-derived sedentary time (min/day)  **Physical activity**  Accelerometer-derived LPA (min/day), MPA (min/day), VPA (min/day) | Physical fitness (Cardiorespiratory fitness: 20 metre shuttle run; muscular fitness: handgrip strength, standing long jump, and speed-agility: 4x10 metre shuttle run) objectively measured using the PREFIT fitness test battery. | Fitness | Replacing 5 min of LPA with 5 min of sedentary time was not associated with 20 metre shuttle (β=0.05; 95%CI: -0.03,0.12), handgrip strength (β=0.00; 95%CI: -0.04,0.05), or 4 x10 metre shuttle (β=-0.03; 95%CI: -0.08,0.03); but was positively associated with standing long jump (β=0.46; 95%CI: 0.02,0.90).  Replacing 5 min of MPA with 5 min of sedentary time was not associated with any fitness indicator (20 metre shuttle (β=-0.01; 95%CI: -0.10,0.08), handgrip strength (β=-0.03; 95%CI: -0.09,0.02), standing long jump (β=-0.40; 95%CI:- 0.94,0.14), or 4 x10 metre shuttle (β=0.00; 95%CI: -0.07,0.07).  Replacing 5 min of VPA with 5 min of sedentary time was not associated handgrip strength (β=-0.17; 95%CI: -0.38,0.04); was negatively associated with 20 metre shuttle (β=-0.87; 95%CI: -1.21,-0.53) and standing long jump (β=-2.12; 95%CI: -4.20,-0.04); and was associated with a slower 4 x10 metre shuttle (β=0.62; 95%CI: 0.36,0.88). |

95%CI = 95% confidence interval; LPA = light-intensity physical activity; min = minutes; MPA = moderate-intensity physical activity; PA = physical activity; VPA = vigorous-intensity physical activity.

**Table S4. Summary of included studies for growth**

| Refid #. Author (year); country | Study Design | Sample | Exposure | Indicator | Indicator Category | Main Findings |
| --- | --- | --- | --- | --- | --- | --- |
| 26. Goldfield et al. (2016) [27]. Canada | Clustered RCT | n=83; mean age: 3.3 years | Intervention (6 months):  **Sedentary Behaviour:**  In addition to the PA component of intervention, parents were given the Canadian Society for Exercise Physiology Sedentary Behaviour Guidelines for the early years.  **Physical Activity**:  Two 3-hour training workshops to child care providers delivered by an early learning PA specialist, a resource training manual, and some basic equipment (eg, balls, skipping ropes). Training binders included binders included physical activity guidelines, recommended weekly activity program and logsheet, as well as plans for bad weather.  In addition there were 12, 1 hour "booster" sessions provided biweekly.  Control:  Wait list control continued to provide their standard childcare curriculum during the study period. | Objective height and weight measurements. | Adiposity | Intervention was not associated with changes in height (Mean group x time difference= 0.01m ; 95%CI: -0.004, 0.006; p=0.794)or weight (Mean group x time difference= -0.0kg ; 95%CI: -0.4, 0.1; p=0.299).  Note: Significant group x time interaction for TPA (Control: -3.1 min/day; intervention: 19.4 min/day), LPA (Control: -8.3 min/day; intervention: 7.8 min/day), and sedentary time (Control: 10.2 min/day; intervention: -12.3 min/day). No group x time interaction for MVPA (Control: 5.3 min/day; intervention: 11.8 min/day, p=0.085). |
| 11. Ip et al. (2016) [30]. USA | Longitudinal | n=248; Age range: 2.5-3.5 years | **Sedentary Behaviour:**  Accelerometer-derived minutes/day sedentary time  **Physical Activity:**  average total minutes of  light activity per day, average minutes per bout of MVPA, average MET score per MVPA bout, total minutes of MVPA per day, total bouts of MVPA per day, standard deviation of total MVPA bouts per day, standard deviation of average MET score per MVPA bout.  **Combination:**  All of these variables used (as well as autocorrelation between observations) to create less active and more active groups using hidden Markov modeling. | Objective weight measurement transformed into CDC percentiles. | Growth | Belonging to the more active group was not associated with weight percentile over the 2 year duration of the study (β= -0.39; SE=1.24; p=0.76).  Note: More active state was associated with higher LPA (total bouts per day (p<0.01), average minutes per bout (p<0.001), average MET score per bout (p<0.001), and total minutes per day (p=0.01)), MVPA (total bouts per day (p<0.001), average minutes per bout (p<0.001), average MET score per bout (p<0.001), and total minutes per day (p<0.001)), but not with minutes per day sedentary (p=0.09) which was the only sedentary variable. |

95%CI = 95% confidence interval; CDC = Center for Disease Control; LPA = light-intensity physical activity; MET = metabolic equivalent; min = minutes; MVPA = moderate- to vigorous-intensity physical activity; PA = physical activity; RCT = randomized controlled trial; SE = standard error; TPA = total physical activity.
